# Supplementary material for: An optimized quantitative proteomics method establishes the cell type‐resolved mouse brain secretome
Source: EMBO J. 2020 Sep 21;39(20):e105693. doi: 10.15252/embj.2020105693 (PMC7560198; doi:10.15252/embj.2020105693)
Supplement: Supplementary file 1 — Appendix [file EMBJ-39-e105693-s001.pdf]

# APPENDIX

## Tables of contents


1. Appendix Figure S1:
Diversity of cell type-resolved mouse brain glyco-secretome resource.

2. Appendix Figure S2:
Top25 enriched proteins in the secretome of astrocytes, microglia, neurons and oligodendrocytes.

3. Appendix Table S1:
List of BACE1 substrate candidates.

**Appendix Fig S1: Diversity of cell type-resolved mouse brain glyco-secretome resource**

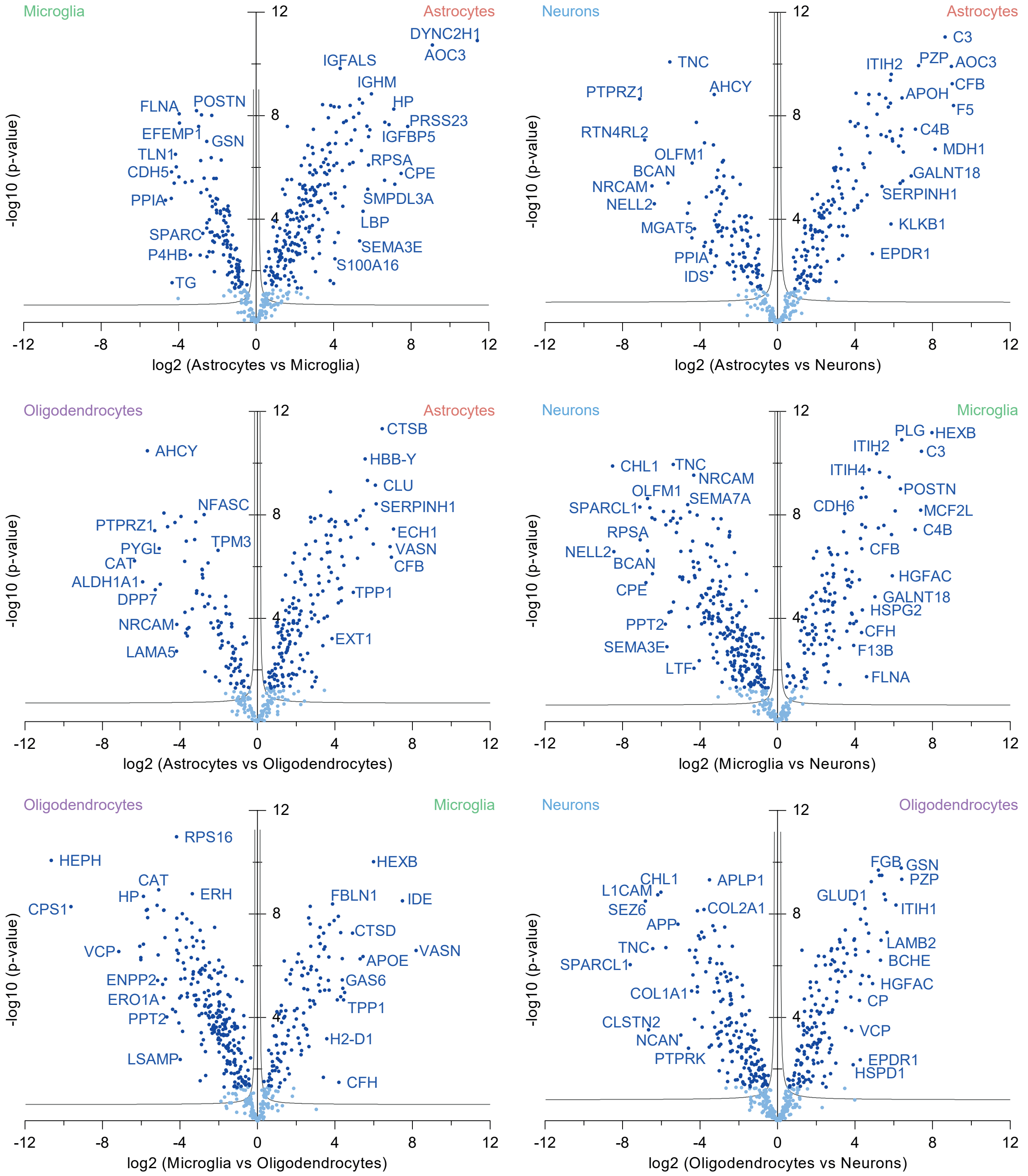


Pairwise comparison of the secretomes of the four different cell types. Volcano plot indicating the proteins in the conditioned media of the different primary brain cells analyzed with the hiSPECS DIA method (N=6). The negative log10 transformed p-value of each protein is plotted against its log2 fold change comparing all investigated cell types with each other. Significantly regulated proteins (p-value< 0.05) are indicated in dark blue. The grey hyperbolic curves depict a permutation based false discovery rate estimation (p = 0.05; s0 = 0.1).

**Appendix Fig S2: Top25 enriched proteins in the secretome of astrocytes, microglia, neurons and oligodendrocytes.**

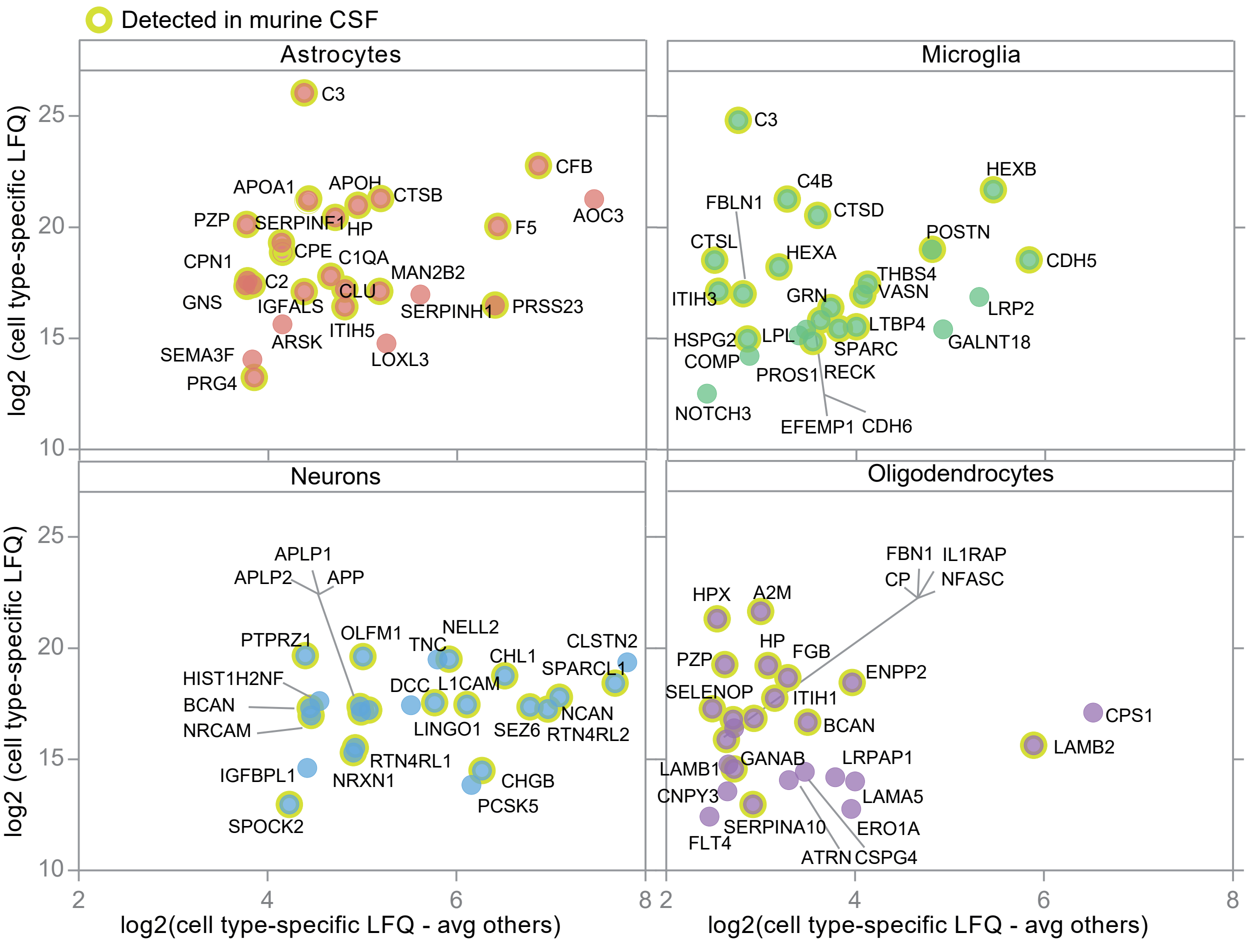


Representation of the top 25 glycoproteins enriched in the secretome of one cell type. The average log2 LFQ intensities are plotted against the log2 LFQ ratios of the cell type specific-abundance subtracted from the average of the other cell types. Therefore, the values on the y-axis roughly indicate the abundance within a cell´s secretome, whereas the values on the x-axis show the enrichment compared to the other cell types in log2 scale. Yellow circles indicate proteins which are also detected in murine CSF. One example is HEXB in the microglia panel with an average LFQ value of 21.2 (y-axis) and a value of 5.4 on the x-axis. This indicates that the protein has a $2^{5.4}$-fold higher level in the secretome of microglia compared to the average of its levels in the secretome of the other three cell types.

**Table S1: List of BACE1 substrate candidates.**

Summary of the significantly reduced proteins (p-values < 0.05) in the secretome of neurons upon pharmacological inhibition of BACE1 with C3 identified by the hiSPECS DIA method (Fig 5). Listed are the gene names, UniProt accession, ratio between C3 vs control samples (DMSO), p-value and the topology of the proteins. Other proteomic studies are highlighted which previously showed a reduction of the proteins upon BACE1 inhibition in primary neurons or murine CSF.

| Gene name | UniProt Ac. | T-test p-value | Ratio (C3/Ctr) | Topology | Publication |
|---|---|---|---|---|---|
| Sez6 | Q7TSK2 | 3.04E-07 | 0.06 | TM1 | Kuhn et al., 2012 |
| Adam22 | Q9R1V6 | 2.55E-15 | 0.08 | TM1 | |
| Cd200 | O54901 | 1.04E-21 | 0.11 | TM1 | |
| Sez6l | Q6P1D5 | 4.25E-06 | 0.14 | TM1 | Kuhn et al., 2012 |
| Cxadr | P97792 | 2.83E-13 | 0.26 | TM1 | |
| Il6st | Q00560 | 2.54E-03 | 0.27 | TM1 | |
| Aplp1 | Q03157 | 1.60E-08 | 0.28 | TM1 | Kuhn et al., 2012; Zhou et al., 2012; Dislich et al., 2015 |
| Cachd1 | Q6PDJ1 | 1.46E-12 | 0.33 | TM1 | Kuhn et al., 2012 |
| Sema4a | Q62178 | 2.17E-07 | 0.34 | TM1 | |
| Plxdc2 | Q9DC11 | 4.07E-12 | 0.37 | TM1 | Kuhn et al., 2012; Zhou et al., 2012; Dislich et al., 2015 |
| Alcam | Q61490 | 1.37E-05 | 0.40 | TM1 | |
| Lrrn1 | Q61809 | 2.65E-12 | 0.42 | TM1 | Kuhn et al., 2012 |
| Cntn2 | Q61330 | 8.23E-14 | 0.47 | GPI | Kuhn et al., 2012; Zhou et al., 2012; Dislich et al., 2015 |
| Mmp24 | Q9R0S2 | 4.96E-05 | 0.55 | TM1 | |
| Glg1 | Q61543 | 5.19E-10 | 0.56 | TM1 | Kuhn et al., 2012,9 |
| Fgfr1 | P16092 | 4.61E-05 | 0.57 | TM1 | Kuhn et al., 2012; Zhou et al., 2012; Dislich et al., 2015 |
| Vcam1 | P29533 | 1.13E-07 | 0.59 | TM1 | |
| Chl1 | P70232 | 1.64E-09 | 0.60 | TM1 | Kuhn et al., 2012; Zhou et al., 2012; Dislich et al., 2015 |
| Sort1 | Q6PHU5 | 1.25E-04 | 0.60 | TM1 | |
| Sema4b | Q62179 | 3.17E-07 | 0.70 | TM1 | Kuhn et al., 2012 |
| Col4a1 | P02463 | 2.50E-03 | 0.75 | | |
| Itm2b | O89051 | 1.77E-02 | 0.78 | TM2 | |
| Met | P16056 | 7.93E-06 | 0.80 | TM1 | |
| Col4a2 | P08122 | 1.72E-03 | 0.81 | | |
| Robo1 | O89026 | 4.41E-03 | 0.82 | TM1 | |
| Adam12 | Q61824 | 4.85E-02 | 0.82 | TM1 | |
| Tyro3 | P55144 | 5.02E-02 | 0.83 | TM1 | |
| Tnfrsf21 | Q9EPU5 | 1.62E-02 | 0.84 | TM1 | |
| Sema6c | Q9WTM3 | 7.94E-04 | 0.85 | TM1 | |
| St6galnac 5 | Q9QYJ1 | 4.80E-02 | 0.86 | TM2 | |
| Mdga1 | Q0PMG2 | 2.57E-03 | 0.86 | GPI | |
| Ptprg | Q05909 | 9.09E-04 | 0.88 | TM1 | |
| Rgma | Q6PCX7 | 3.51E-02 | 0.91 | GPI | |
