# Supplementary material for: An optimized quantitative proteomics method establishes the cell type‐resolved mouse brain secretome
Source: EMBO J. 2020 Sep 21;39(20):e105693. doi: 10.15252/embj.2020105693 (PMC7560198; doi:10.15252/embj.2020105693)
Supplement: Supplementary file 2 — Expanded View Figures PDF [file EMBJ-39-e105693-s002.pdf]

Expanded View Figures

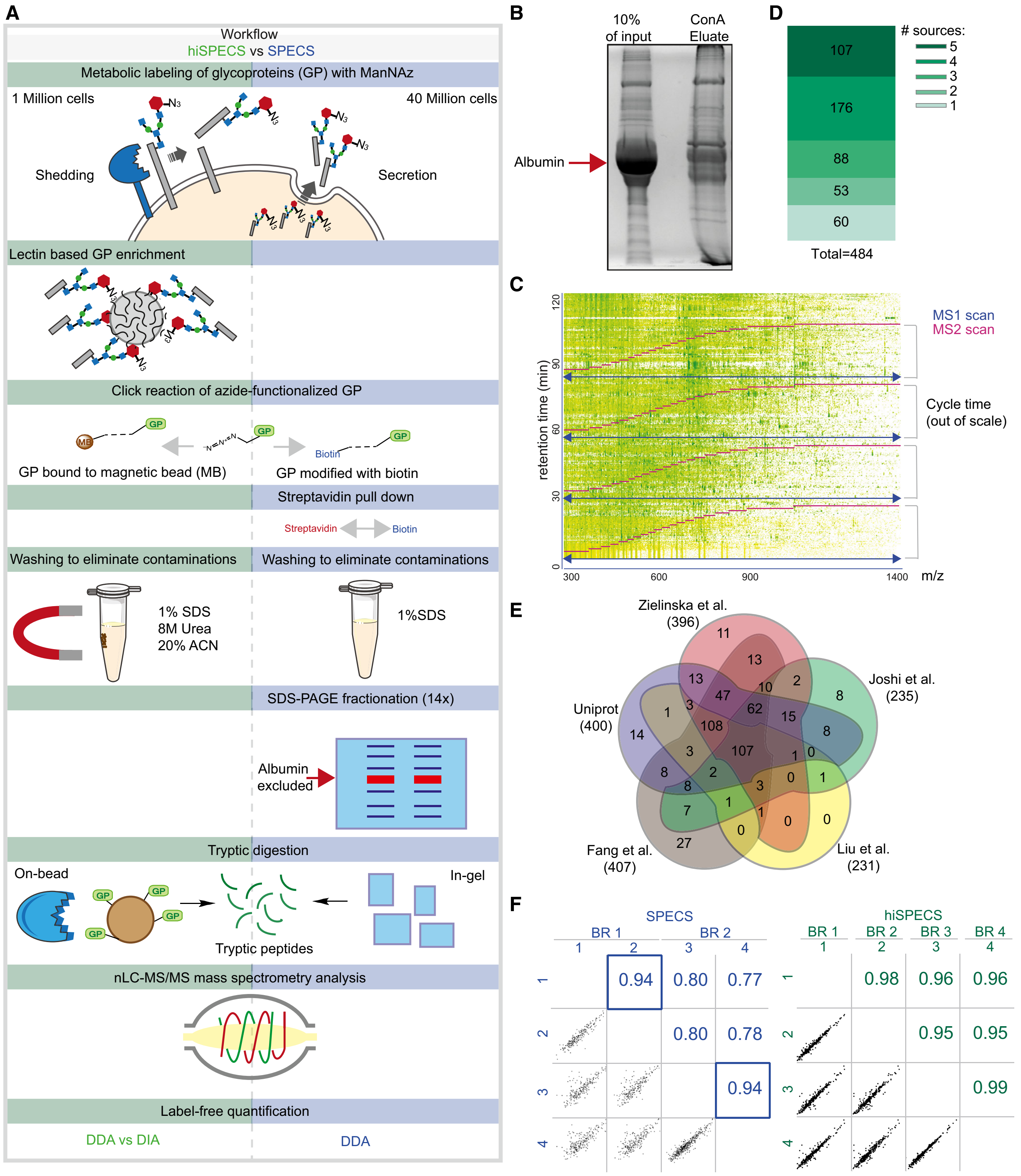

Figure EV1.

◀

Figure EV1. Benchmarking hiSPECS against SPECS.

A Comparison of the novel hiSPECS (green) and the previous SPECS (blue) protocol (Kuhn *et al*, 2012). hiSPECS uses lectin-based glycoprotein enrichment followed by covalent binding to magnetic beads which improves sample processing. On-bead tryptic digestion is followed by mass spectrometry analysis and label-free quantification (LFQ) using data-independent or data-dependent acquisition (DIA vs DDA). In contrast, SPECS depends on biotinylation of the azide-functionalized glycoproteins, followed by streptavidin pull-down, SDS-gel-based fractionation into 14 gel slices, and DDA analysis only.

B Coomassie-stained gel showing the prominent reduction in albumin as a result of glycoprotein enrichment with ConA. Left lane: secretome (conditioned medium) of primary neurons before glycoprotein enrichment. 10% of the volume was loaded that was used for the ConA enrichment. Right lane: eluate after glycoprotein enrichment using ConA beads. The albumin band is highlighted with a red arrow.

C A representative peptide density plot of a neuronal secretome using the hiSPECS method and analyzed in DDA mode. The *m/z* ratio is plotted against the retention time. One MS1 scan (purple) is followed by 20 MS2 scans covering a range of 300–1,400 *m/z* with an overlap of 1 *m/z* between adjoining *m/z* windows. The *m/z* windows were adjusted to achieve equal numbers of peptides.

D Identified glycoproteins in the secretome of primary neurons using the DIA hiSPECS protocol according to UniProt, (Fang *et al*, 2016), (Joshi *et al*, 2018), (Liu *et al*, 2017), or (Zielinska *et al*, 2010).

E Venn diagram illustrating in which sources the proteins of (D) were found to contain a glycosylation site. As expected for the glyco-secretome, more than 85% of the quantified secretome proteins were annotated as glycoproteins.

F Representative Pearson correlations of log_2-transformed protein LFQ intensities of four biological replicates (BR) processed with either the SPECS or hiSPECS method. In the previous SPECS studies, sample pairs were separated on the same gel to achieve high reproducibility, whereas the correlation of biological replicates run on different gels was rather low. Thus, protein LFQ ratios of the individual replicates were used for statistical evaluation. The blue squares indicate samples run on the same gel during sample preparation [SPECS data obtained from (Kuhn *et al*, 2012)].

 © 2020 The Authors

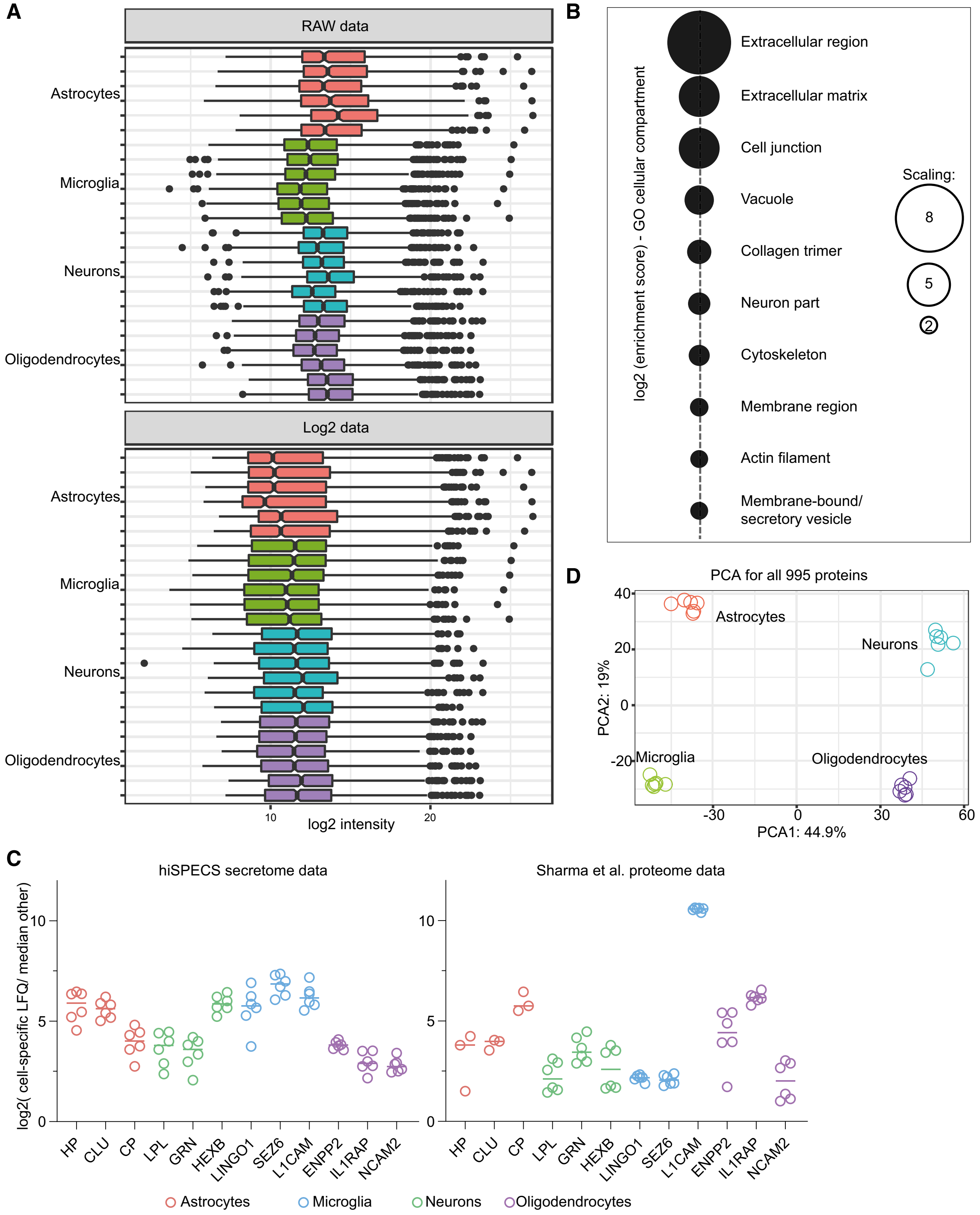
A
RAW data
Log2 data
Astrocytes
Microglia
Neurons
Oligodendrocytes
10
20
log2 intensity
B
log2 (enrichment score) - GO cellular compartment
Extracellular region
Extracellular matrix
Cell junction
Vacuole
Collagen trimer
Neuron part
Cytoskeleton
Membrane region
Actin filament
Membrane-bound/
secretory vesicle
Scaling:
8
5
2
D
PCA for all 995 proteins
PCA2: 19%
PCA1: 44.9%
Astrocytes
Neurons
Microglia
Oligodendrocytes
C
hiSPECS secretome data
Sharma et al. proteome data
log2(cell-specific LFQ/ median other)
HP
CLU
CP
LPL
GRN
HEXB
LINGO1
SEZ6
L1CAM
ENPP2
IL1RAP
NCAM2
Astrocytes
Microglia
Neurons
Oligodendrocytes

Figure EV2.

Figure EV2. Quality control of cell type-resolved mouse brain secretome resource.

A Data transformation of the hiSPECS DIA secretome analysis of the brain cell types. Log_2 transformation of the intensities (N = 1). The central band indicates the median, the boxes indicate the lower or upper quartile, the whiskers indicate minimum or maximum, and dots indicate outliers.

B Functional annotation clustering with DAVID 6.8 (da Huang *et al*, 2009a; da Huang *et al*, 2009b) for gene ontology term cellular component (FAT) of the 995 hiSPECS proteins identified using the mouse proteome as background. The dot sizes indicate the log_2 enrichment score.

C Fold change in cell type-specific proteins in the brain cells. Log_2 ratio of the average abundance in the specific cell type to the median abundance in the other cell types in the hiSPECS secretome or lysate analysis (Sharma *et al*, 2015) is shown. Known cell type-specific marker proteins are highlighted for each cell type which reveals a strong enrichment in the lysate and secretome of the primary brain cells verifying the quality and comparability of the primary cultures. For example, the ectodomain of the membrane protein NCAM2 (with an *y*-axis value of 3 in the log_2 scale) is secreted about eightfold more from oligodendrocytes compared with the median of the other three cell types and also expressed at a higher level in this cell type compared with the median of the other three cell types. Horizontal lines indicate the mean.

D Principal component analysis (PCA). The secretomes of the four cell types segregated based on the two major components of all 995 proteins identified in at least 5 biological replicates in one cell type, which accounted for 44.9% and 19% of the variability, respectively.

© 2020 The Authors

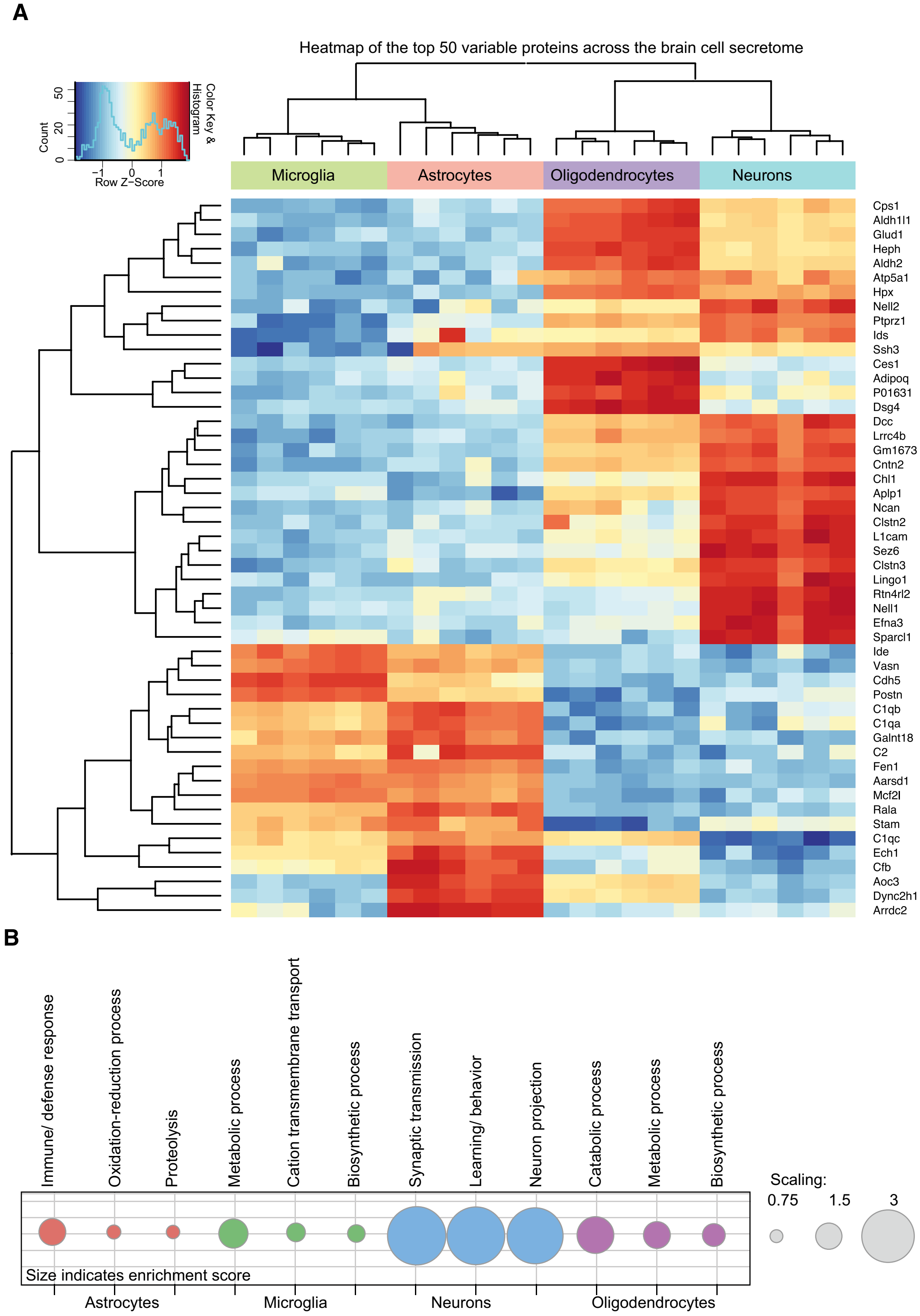
A
Heatmap of the top 50 variable proteins across the brain cell secretome
Color Key & Histogram
Count
Row Z−Score
Microglia
Astrocytes
Oligodendrocytes
Neurons
Cps1
Aldh1l1
Glud1
Heph
Aldh2
Atp5a1
Hpx
Nell2
Ptprz1
Ids
Ssh3
Ces1
Adipoq
P01631
Dsg4
Dcc
Lrrc4b
Gm1673
Cntn2
Chl1
Aplp1
Ncan
Clstn2
L1cam
Sez6
Clstn3
Lingo1
Rtn4rl2
Nell1
Efna3
Sparcl1
Ide
Vasn
Cdh5
Postn
C1qb
C1qa
Galnt18
C2
Fen1
Aarsd1
Mcf2l
Rala
Stam
C1qc
Ech1
Cfb
Aoc3
Dync2h1
Arrdc2
B
Immune/ defense response
Oxidation-reduction process
Proteolysis
Metabolic process
Cation transmembrane transport
Biosynthetic process
Synaptic transmission
Learning/ behavior
Neuron projection
Catabolic process
Metabolic process
Biosynthetic process
Scaling:
0.75
1.5
3
Size indicates enrichment score
Astrocytes
Microglia
Neurons
Oligodendrocytes

Figure EV3.

◀ **Figure EV3. Top 50 cell type-specifically enriched proteins in the secretome resource.**

A Heat map of the top 50 differentially secreted proteins (Bonferroni's *P*.adj < 0.05 using the R package Limma (Ritchie *et al*, 2015)) across the four cell types from hierarchical clustering. For the missing protein quantification data, an imputation approach was undertaken using data missing at random within a left-shifted Gaussian distribution by 1.8 standard deviation. The rows represent the differentially secreted proteins, and the columns represent the cell types with their replicates. The colors represent log-scaled protein levels with blue indicating the lowest, white indicating intermediate, and red indicating the highest protein levels.

B Functional annotation clustering with DAVID 6.8 (da Huang *et al*, 2009a; da Huang *et al*, 2009b) for gene ontology term biological process (FAT) of the cell type-specific secretome proteins (Table EV4). All proteins detected in the hiSPECS brain secretome study have been chosen as the background. The dot sizes indicate the enrichment score.

 © 2020 The Authors

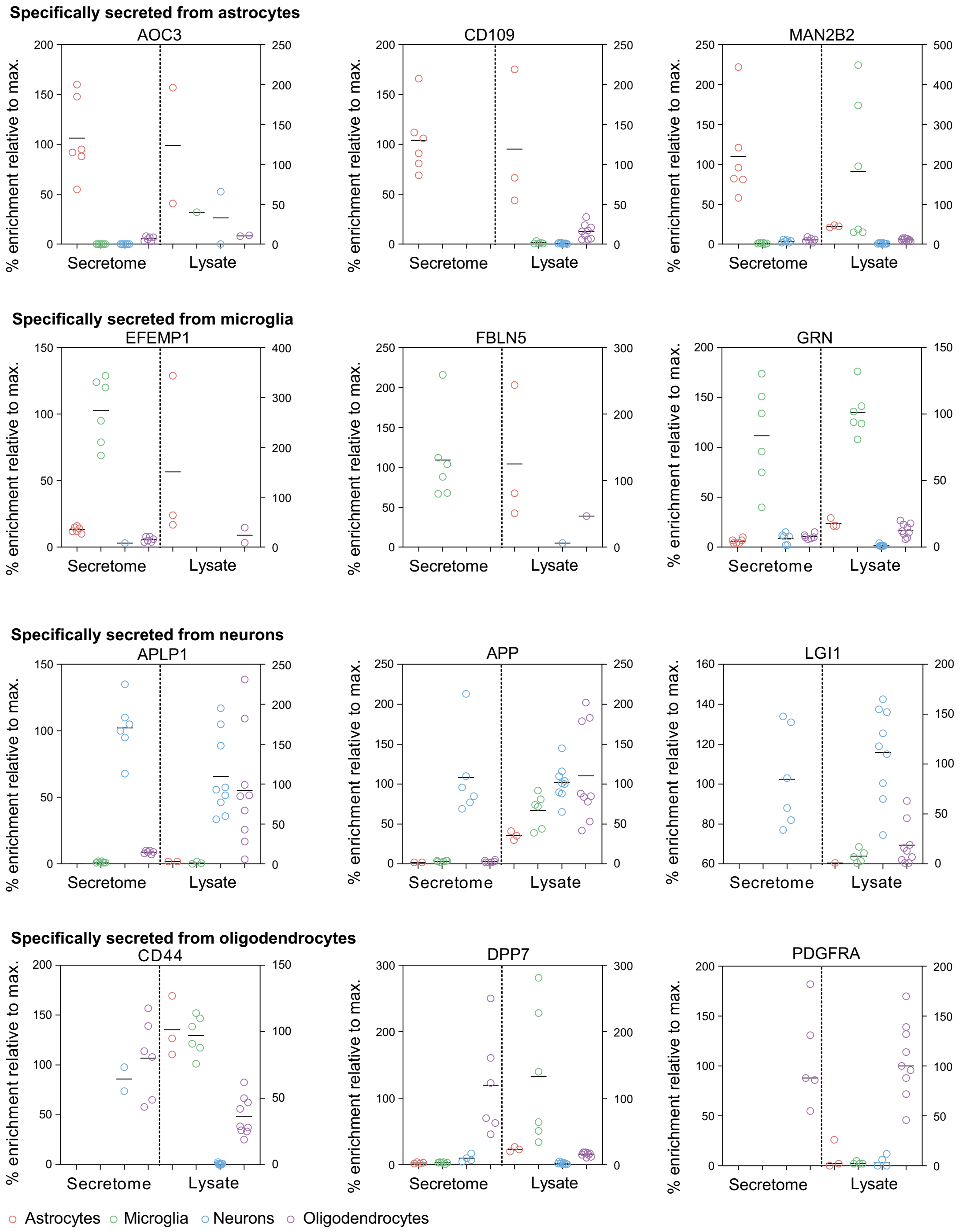

Figure EV4. Protein levels in the brain cell secretome vs lysate proteome.

Comparison of the iSPECS secretome resource and lysate data by Sharma *et al* (2015). The % enrichment is indicated normalized to the average of the most abundant cell type. For example, APLP1 is similarly abundant in lysates of neurons and oligodendrocytes, but only secreted to a relevant extent from neurons. Thus, APLP1 is classified as a cell type-specifically secreted protein. Horizontal lines indicate the mean.

Figure EV5. Substrate candidate identification of the Alzheimer's protease BACE1 as proof of principle of the hiSPECS method.

- A The same experiment as in Fig 5A and B, but using DDA instead of DIA for mass spectrometric data acquisition. The volcano plot shows changes in protein levels in the secretome of primary cultured neurons (hip + ctx) upon BACE1 inhibitor C3 treatment using the hiSPECS DDA method. The negative log_{10}-transformed *P*-value (two-sample *t*-test) of each protein is plotted against its log_2 fold change comparing inhibitor-treated and control condition ($N = 11$). The gray hyperbolic curves depict a permutation-based false discovery rate estimation ($P = 0.05$; $s0 = 0.1$). Significantly regulated proteins ($P < 0.05$) are indicated with a dark blue dot, known BACE1 substrates are indicated with blue letters, and unknown ones are indicated with black letters. The two newly validated BACE1 substrates CD200 and ADAM22 are indicated in red. SEZ6 and IL6ST are not depicted in the volcano plot because they were only identified in the control condition.
- B Correlation of the hiSPECS DDA to DIA method. Plotted are the log_2 fold changes between C3 treatment and DMSO control samples ($N = 11$). The two newly validated BACE1 substrates CD200 and ADAM22 are indicated in red, known BACE1 substrates in blue, and unknown ones in black.
- C The volcano plot (details described in (A)) shows changes in protein levels in the secretome of primary cultured neurons isolated either from the hippocampus (hip) or from cerebral cortex (ctx) using the hiSPECS DIA method. Proteins exclusively detected in one group are indicated.
- D Correlation analysis of the average log_2 LFQ values of the secretome proteins of (C).
- E Venn diagram indicating the overlap of quantified proteins in the secretome of hippocampal vs cortical neurons. Proteins were considered robustly quantified if detected with at least 5 of 6 biological replicates.
- F The same experiment as (A), however, using only hippocampal neurons and hiSPECS DIA ($N = 8$). Proteins were considered if quantified in at least 6 of 8 biological replicates in one group.
- G Correlation of the hiSPECS DIA method comparing hippocampal to mainly cortical (mixed ctx and hip, as described in legend to panel A neuronal secretome; Fig 5B). Plotted are the log_2 fold changes between C3 treatment and DMSO control samples. The newly validated BACE1 substrate CD200 is indicated in red, known BACE1 substrates in blue, and unknown ones in black.

 © 2020 The Authors

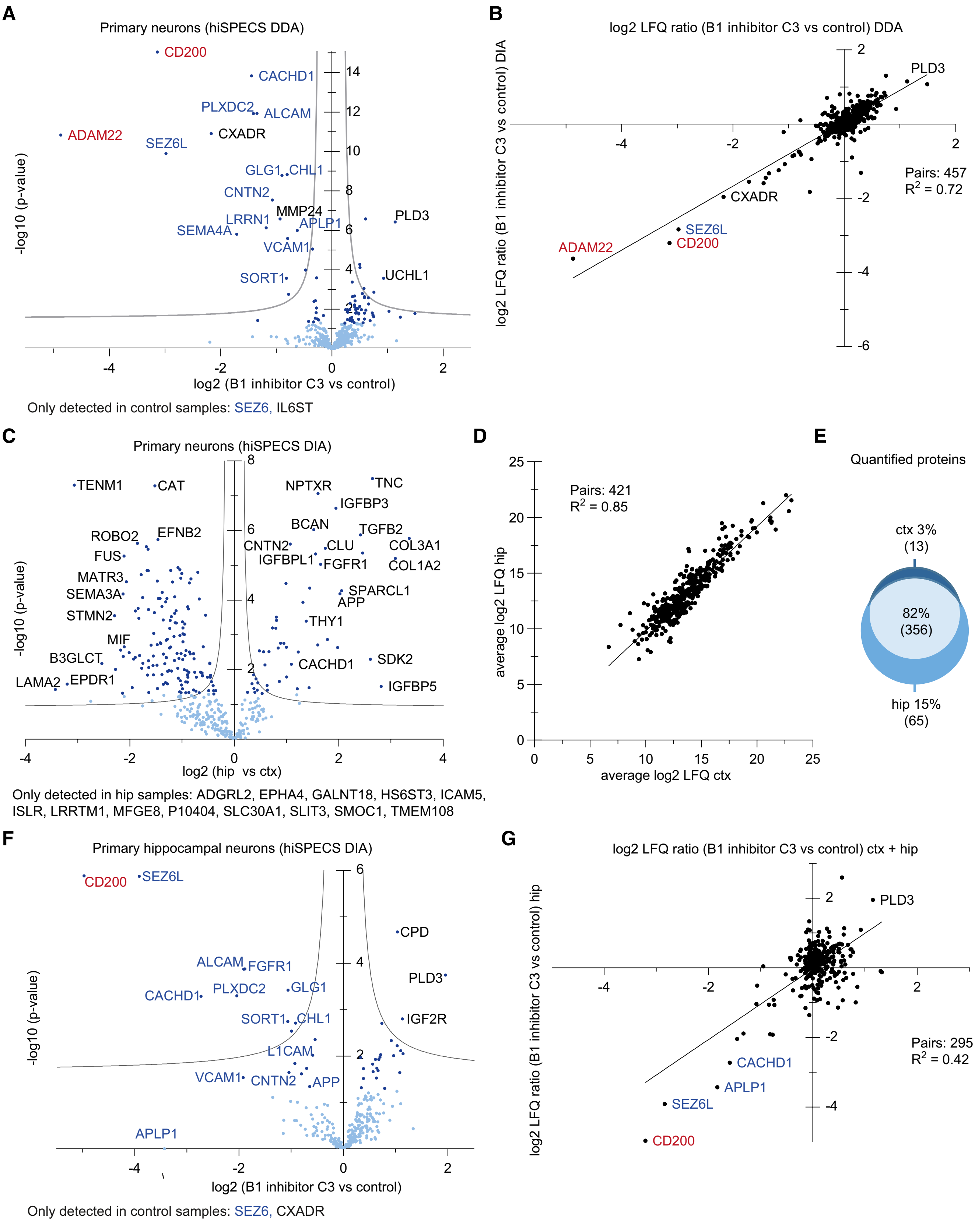
A
Primary neurons (hiSPECS DDA)
CD200
CACHD1
PLXDC2
ALCAM
ADAM22
CXADR
SEZ6L
GLG1
CHL1
CNTN2
MMP24
LRRN1
APLP1
PLD3
SEMA4A
VCAM1
SORT1
UCHL1
-log10 (p-value)
log2 (B1 inhibitor C3 vs control)
Only detected in control samples: SEZ6, IL6ST
B
log2 LFQ ratio (B1 inhibitor C3 vs control) DDA
log2 LFQ ratio (B1 inhibitor C3 vs control) DIA
PLD3
CXADR
SEZ6L
CD200
ADAM22
Pairs: 457
R2 = 0.72
C
Primary neurons (hiSPECS DIA)
TENM1
CAT
NPTXR
TNC
IGFBP3
ROBO2
EFNB2
BCAN
TGFB2
CNTN2
CLU
COL3A1
FUS
IGFBPL1
FGFR1
COL1A2
MATR3
SEMA3A
SPARCL1
APP
STMN2
THY1
MIF
B3GLCT
SDK2
CACHD1
LAMA2
EPDR1
IGFBP5
-log10 (p-value)
log2 (hip vs ctx)
Only detected in hip samples: ADGRL2, EPHA4, GALNT18, HS6ST3, ICAM5, ISLR, LRRTM1, MFGE8, P10404, SLC30A1, SLIT3, SMOC1, TMEM108
D
Pairs: 421
R2 = 0.85
average log2 LFQ hip
average log2 LFQ ctx
E
Quantified proteins
ctx 3%
(13)
82%
(356)
hip 15%
(65)
F
Primary hippocampal neurons (hiSPECS DIA)
CD200
SEZ6L
CPD
ALCAM
FGFR1
PLD3
CACHD1
PLXDC2
GLG1
SORT1
CHL1
IGF2R
L1CAM
VCAM1
CNTN2
APP
APLP1
-log10 (p-value)
log2 (B1 inhibitor C3 vs control)
Only detected in control samples: SEZ6, CXADR
G
log2 LFQ ratio (B1 inhibitor C3 vs control) ctx + hip
log2 LFQ ratio (B1 inhibitor C3 vs control) hip
PLD3
CACHD1
APLP1
SEZ6L
CD200
Pairs: 295
R2 = 0.42

Figure EV5.
