# Supplementary figures and images for: An optimized quantitative proteomics method establishes the cell type‐resolved mouse brain secretome
Source: EMBO J. 2020 Sep 21;39(20):e105693. doi: 10.15252/embj.2020105693 (PMC7560198; doi:10.15252/embj.2020105693)

# Source data

EMBOJ-2020-105693\_Tueshaus et al.

Figure 5C

Figure 5C

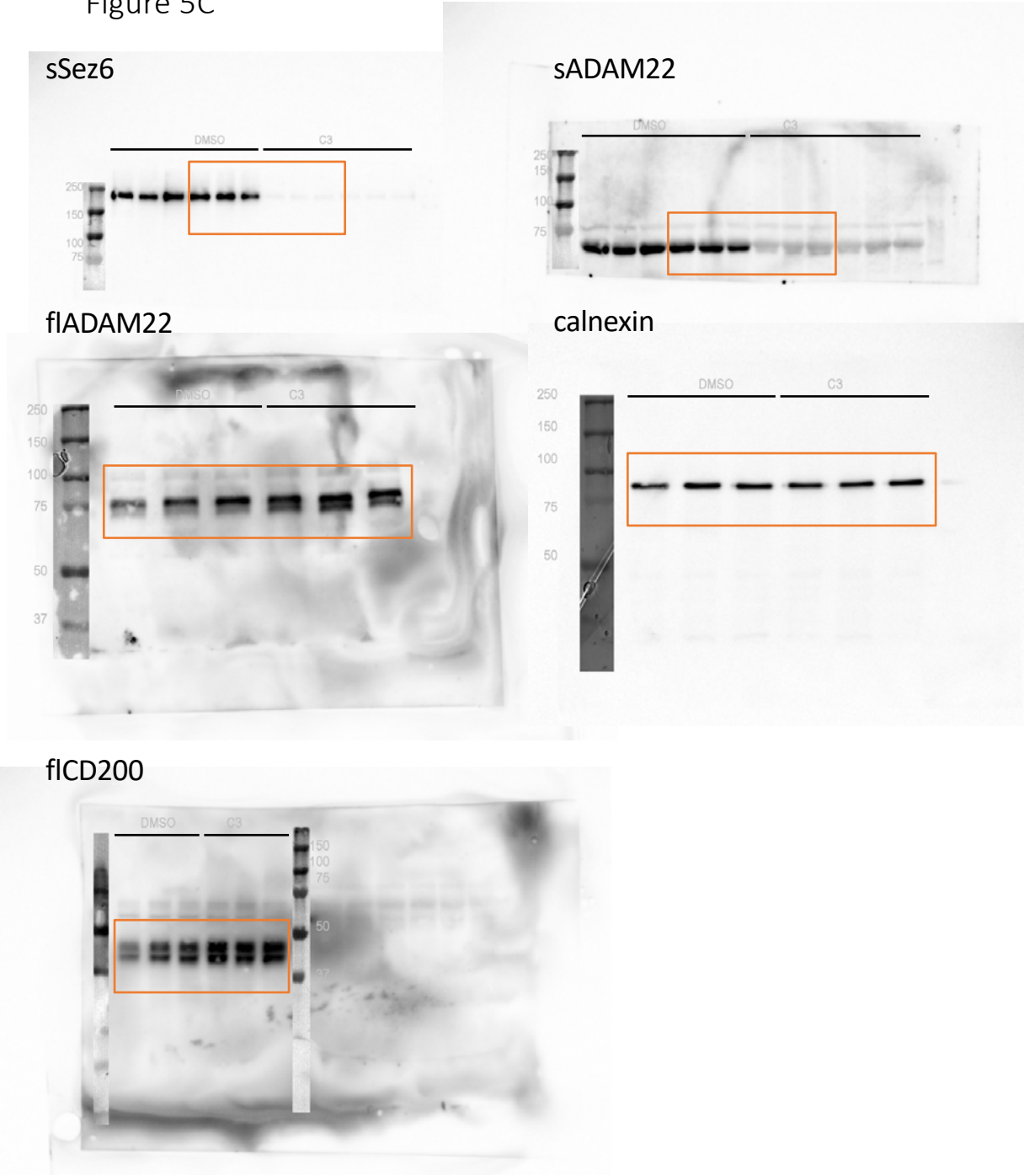

sSez6

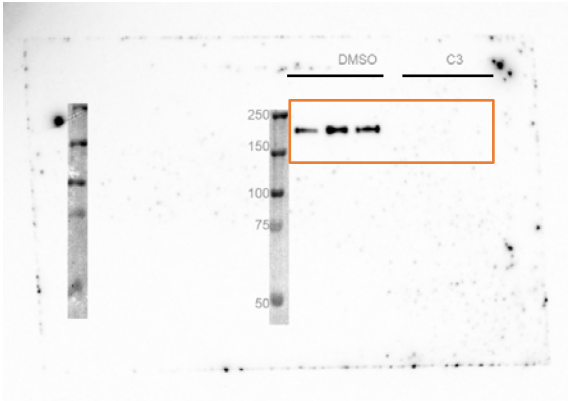

sADAM22

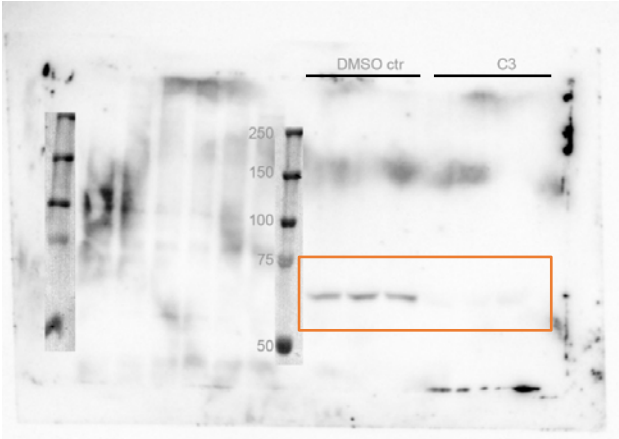

flADAM22

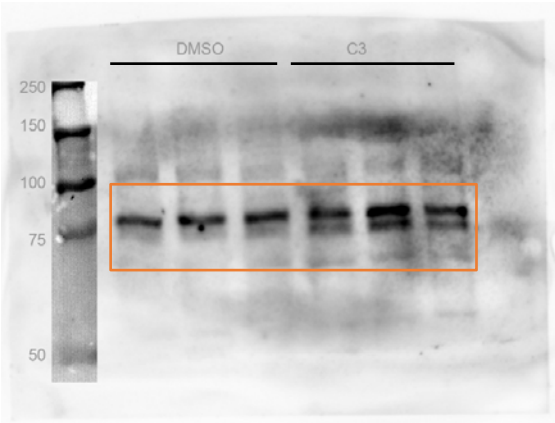

calnexin

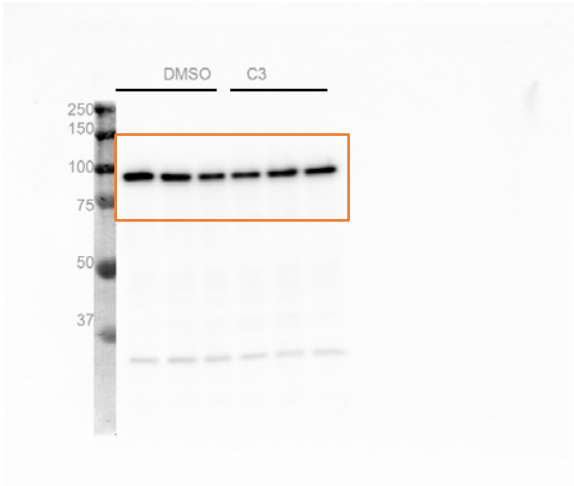

flCD200

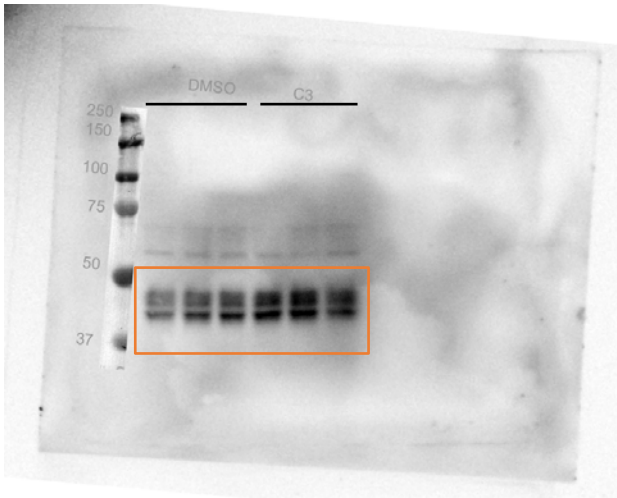

Supplement: Supplementary file 12 — Source Data for Figure 5 [file EMBJ-39-e105693-s011.pdf]
